# Supplementary material for: COVID-19 outcomes in hospitalized puerperal, pregnant, and neither pregnant nor puerperal women
Source: PLoS One. 2021 Nov 15;16(11):e0259911. doi: 10.1371/journal.pone.0259911 (PMC8592461; doi:10.1371/journal.pone.0259911)
Supplement: Attachment — Submitted filename: Response to Reviewers.docx [file pone.0259911.s002.docx]

**Responses to the Reviewers:**

**Reviewer #1: Minor issues:**

**1. Title: is still difficult to understand. “a population study” provides no additional meaning.**

Response: Thank you for your comment. We agree with you. We decided to exclude “a population study” from the title and keep as “COVID-19 outcomes in hospitalized puerperal, pregnant, and neither pregnant nor puerperal women”.

**2. Abstract:
Please separate factor matched for from outcomes.
It is not clear if the outcomes mentioned in the abstract are after propensity matching.**

Response: Thank you for your comment. The outcomes results mentioned in the abstract are obtained after propensity matching and this information is presented in the current version of the manuscript.

**3. The discussion should provide some hypothesis why puerperal women had poorer outcomes.**

Response: Thank you for this comment as well. We added more hypotheses in the manuscript, also written below:

“Some hypotheses can explain the worst prognosis for postpartum women. The puerperium is considered a period of high risk for the occurrence of thromboembolism, such as COVID-19, which can have an additive effect on these occurrences [29,30]. In addition, C-sections increase the risk of maternal mortality, and, in Brazil, more than 55% of births occur through this mode [31,32]. With COVID-19, the risk may be strengthened. Another hypothesis is the three-delays model [33]. During the puerperium, it is common for pregnant women to neglect their own health care because they are focused on taking care of their newborn, reflecting the delay in deciding to seek medical assistance, which is compatible with the higher frequency of O2 saturation lower than 95% at admission when compared to pregnant women [34].”
